# Functional positional eye and eyelid movements

**DOI:** 10.1007/s00415-020-10071-7

**Published:** 2020-07-15

**Authors:** Mohamed Mabrouk Mustafa, Harry Akram, Manuel Oliva-Domínguez, Diego Kaski

**Affiliations:** 1 Department of Neuroscience, Faculty of Medicine, Jordan University of Science and Technology, Irbid, Jordan; 2 Pharmacology Department, Faculty of Medicine and Health Science, Omdurman Islamic University, Omdurman, Sudan; 3 Department of Neuro-Otology, Royal National Ear Nose and Throat Hospital, University College London Hospitals, Huntley Street, London, UK; 4 ENT Department, Hospital Costa del Sol, Marbella, Spain; 5 Department of Clinical and Motor Neurosciences, University College London, 33 Queen Square, London, UK

**Keywords:** Functional, Eye movements, Positional, Eyelid, Psychogenic, Conversion disorder

## Abstract

**Background:**

Positional manoeuvres are an important part of the neurological examination, particularly in patients with vertigo where the presence and characteristics of provoked nystagmus can help differentiate central from peripheral neurological disorders.

**Methods:**

Case series of functional positional eye and eyelid movements.

**Results:**

We report four patients with functional eye and eyelid movements provoked during positional manoeuvres. The range of abnormalities observed included positional convergence spasm, brief functional saccadic oscillations, and excessive positional blinking mimicking upbeat nystagmus. The functional movements described were present on a background of pre-existing peripheral or central nystagmus, or positional vertigo.

**Conclusion:**

Functional positional eye and eyelid movements may co-exist with organic nystagmus that renders an accurate interpretation of the manoeuvre more challenging. A thorough understanding of the clinical features that differentiate these two categories of eye/eyelid movements makes the analysis easier, thus preventing misdiagnosis and avoiding unnecessary investigations.

**Electronic supplementary material:**

The online version of this article (10.1007/s00415-020-10071-7) contains supplementary material, which is available to authorized users.

## Introduction

Positional manoeuvres are an integral component of the neurological assessment of a patient with vertigo, where the presence of positional nystagmus can help differentiate between peripheral [such as benign paroxysmal positional vertigo (BPPV)] and central (such as cerebellar stroke) vestibular disorders. Functional eye and eyelid movements have been observed in patients undergoing visual and ophthalmological assessments [1] but during a positional manoeuvre the eye movement that is being assessed is commonly brief and its interpretation complex (eye movements may be disconjugate, or nystagmus may beat in multiple planes). Misinterpretation of positional eye movements can thus lead to incorrect diagnosis and unnecessary or inappropriate investigations [2]. It becomes therefore of particular importance to correctly disambiguate between pathological eye movements that indicate a peripheral or central vestibular deficit, versus those that are functional. In this brief report, we report four patients with unusual positional eye and eyelid movements that describe the range of functional positional eye movements observed in clinical practice.

**Fig. 1:**
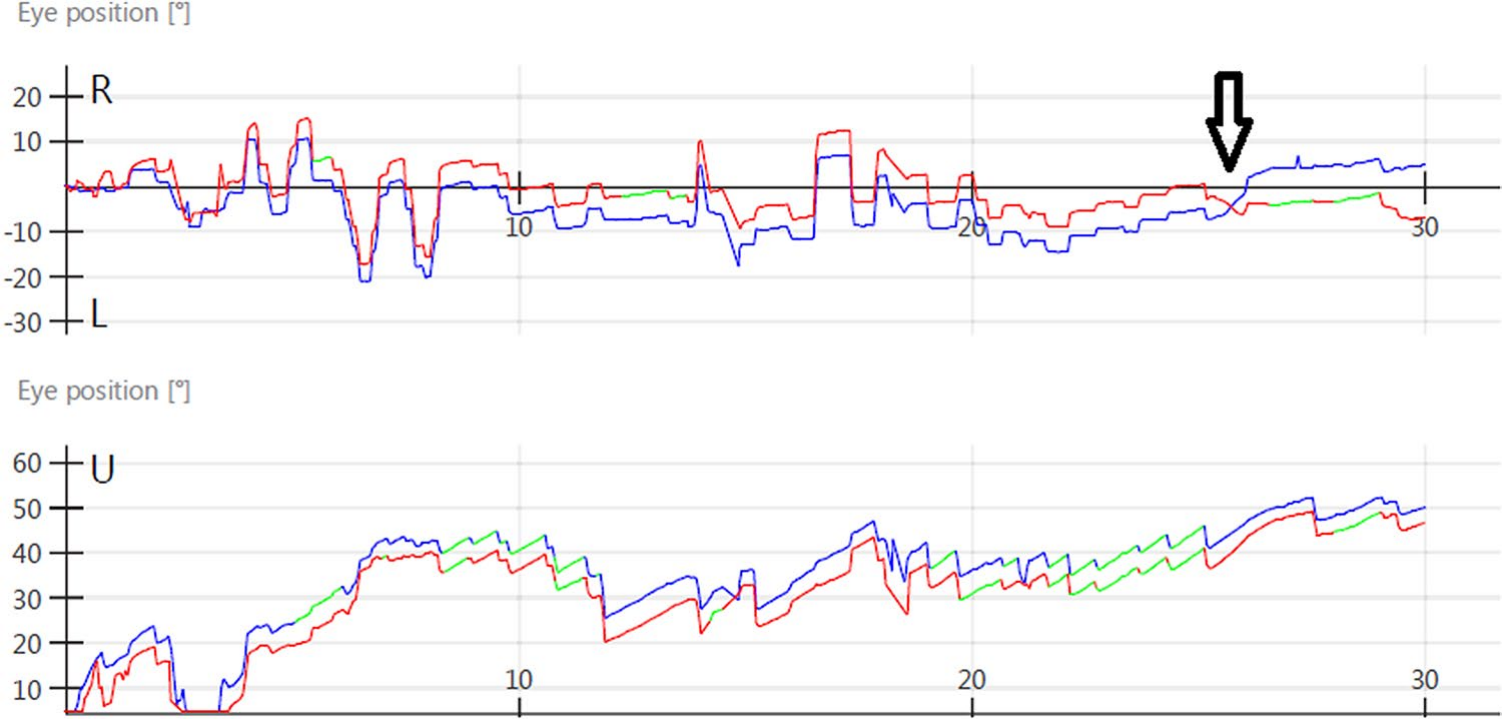
Oculographic study (for case 1), demonstrating positional downbeat nystagmus (note downward deflection of the fast phases in the lower panel, from time = 8 s) in addition to positional convergence at 26 s (black arrow), which occurred upon verbal suggestion by the examiner [note the change of polarity of the left (blue) and right (red) eye traces]. *R* right, *L* left, *U* up

Case 1: a 57-year-old gentleman presented with 8-month history of persistent dizziness and blurring of vision, worsened by walking, watching television, and reading in bed. He had a background of positional downbeat nystagmus that was longstanding (and had never responded to repositioning manoeuvres) thought to be related to the past history of non-Hodgkin’s lymphoma and subsequent chemotherapy. On examination he had positional downbeat nystagmus with associated vertical oscillopsia and mild “fuzziness” of the head and was additionally noted to have convergence during the Dix–Hallpike manoeuvre, occurring more prominently with suggestion and encouragement. The convergent effort was associated with pupillary construction. Pursuit, saccades, and vestibulo-ocular reflex (VOR) were normal. MRI brain, positron emission tomography and cerebrospinal fluid assessment were normal. The positional convergence was thought to be functional in origin, given its intermittent nature, brief duration, and appearance with suggestion (Fig. 1; video 1), on a background of idiopathic (organic) downbeat nystagmus. The functional convergence spasm was no longer present when reviewed 6 months later.

Case 2: a 17-year-old male developed recurrent episodes of spinning vertigo, unsteadiness, headache and nausea, all lasting several minutes, over the previous 6 months. His episodes became prolonged and more frequent, with associated visually-induced dizziness (discomfort and unsteadiness when exposed to visually rich or complex environments) and worsened by being upright or during self-motion. Dix–Hallpike and roll test manoeuvres revealed no nystagmus, but he developed excessive positional blinking during the head-hanging Dix–Hallpike manoeuvre (video 2) during which he described a progressive sensation of rotation that worsened even with the head still in the extended position. The remainder of the examination, and neurological investigations were normal, without any excessive blinking observed. He was diagnosed with persistent postural perceptual dizziness (PPPD) on a background of vestibular migraine, in accordance with the diagnostic criteria from the Barany Society [3]. The excessive blinking persisted at 3 months but subsequently resolved following treatment with cognitive behavioural therapy for PPPD a further 4 months later, that had not explicitly focused on eye movements.

Case 3: a 47-year-old gentleman presented with a prolonged history of episodic rotational vertigo, unsteadiness, oscillopsia, and migrainous headache. Left Dix–Hallpike manoeuvre caused back-to-back saccadic oscillations in the horizontal plane, with a latency of 2–3 s and duration of several seconds. Such movements occurred only during the positional manoeuvre and were reproducible but also highly suppressible with a concomitant cognitive task performed during the Dix–Hallpike manoeuvres. A convergent effort was apparent at onset (video 3). The remainder of the clinical neurological examination and investigations, including MRI brain, were normal. He was diagnosed with vestibular migraine, accounting for the episodic vertigo attacks, and an associated functional positional eye movement disorder. He declined prophylactic migraine treatment with Flunarizine as he had experienced adverse effects from previous migraine medications, and his positional eye movements persisted at 6-month follow-up.

Case 4: a 59-year-old gentleman presented with spinning vertigo, unsteadiness, blurring of vision, and intermittent diplopia. These symptoms increased with head movement. On Dix–Hallpike examination to the right, there was torsional counterclockwise (beating to the patient’s right ear) and up-beating nystagmus followed by a burst of rapid horizontal eye oscillations. Such oscillations stopped on one occasion following instruction to ‘keep the eyes open!’ that appeared to transiently startle the patient. The remainder of the clinical examination was normal. Extensive investigations including MRI brain were normal. He was diagnosed with right-sided BPPV and his symptoms improved with an Epley manoeuvre, such that both the nystagmus and flutter-like oscillations disappeared.

## Discussion

Positional eye movement assessment is an important but perhaps under-utilised component of the eye movement assessment, helping not only in the diagnosis of BPPV, but also a range of central neurological disorders such as Chiari malformation, multiple sclerosis, and multiple system atrophy [4]. Correct identification of positional eye movements can be complicated by extraneous eye and eyelid movements that may occur during the examination. Whilst this may be true of other neuro-ophthalmological assessments [5], in the context of a positional nystagmus, that may be brief, involve complex (sometimes disconjugate) eye oscillations, and may be transient, the ability to differentiate organic (vestibular or cerebellar) eye movements from functional eye/eyelid movements becomes more pertinent.

Convergence spasm remains the only functional positional eye movement that has been reported in the neurological literature [6, 7]. Given the frequency of convergence spasm across outpatient neurology, it is perhaps not surprising that it also appears to be the most commonly observed functional positional eye movement [8, 9]. It can be distinguished from organic causes of ocular esodeviation (most notably sixth nerve palsies) when the convergence is accompanied by pupillary constriction [6], particularly if it occurs with suggestion (case 1, Video 1). The first patient described in this report (case 1) had a background of positional downbeat nystagmus [10], most likely accounting for the persistent symptoms of dizziness and associated visual disturbance with head movement. The positional convergence spasm appeared to be asymptomatic, although if occurring whilst supine may have explained the visual blurring when reading in bed.

In the second case, the patient developed persistent blinking and blepharospasm-like eyelid movements, present only during the positional provocation manoeuvre, and not present during the ‘casual’ examination, again indicative of a functional disorder [11] or a secondary reaction to the provocation manoeuvre. Of note, the frequent blinking induced a recurrent Bell’s phenomenon (upward deviation of the globe occurring during eyelid closure), that could be confused for upbeat nystagmus, or indeed oculogyric crises [12]. The latter however, is typically accompanied by pain, precipitated by use of neuroleptics, and would not be expected to occur exclusively during a positional manoeuvre [13]. Case 2 was diagnosed with PPPD and vestibular migraine, a recognized trigger for PPPD [3], and his symptoms improved with cognitive behavioural therapy for PPPD, and upon repeat examination 4 months after finishing treatment the excessive blinking, and positional vertigo, had disappeared. The temporal relationship between the treatment and resolution of clinical signs suggests that the positional symptoms were either related to PPPD, or more likely associated with vestibular migraine [14], that was now less active.

In cases 3 and 4 we observed horizontal saccadic oscillations mimicking ocular flutter, that are distinguished from this by their brief duration and their modulation by distraction [15]. Moreover, our patient manifest saccadic oscillations only during positional testing—a feature that is not observed in organic flutter. Our patient (case 3) had undergone detailed brain imaging, positron emission tomography and cerebrospinal fluid assessment in relation to his previous lymphoma, but such investigations can be held in abeyance where a thorough examination of the eye movements is suggestive of a functional cause. The symptom of the patient in case 3 fulfilled the criteria for vestibular migraine, and he was offered treatment with Flunarizine. He declined treatment and both symptoms and the positional eye movements persisted at follow-up 6 months later. Whilst in case 3 the positional manoeuvre did not reveal additional organic eye movements, the saccadic oscillations observed in case 4 were accompanied by brief crescendo-decrescendo torsional nystagmus, typical for posterior canal BPPV. We propose that the ensuing saccadic oscillations were a reaction to the BPPV-related positional vertigo, or perhaps an involuntary attempt at suppressing the positional symptoms.

A co-association of organic and functional symptoms or signs is common in neurological disorders [2]. The presence of functional positional eye/eyelid movements with organic nystagmus and vertigo suggests these functional movements may represent an attempt to suppress the underlying nystagmus (and thus reduce the intensity of accompanying vertigo) or be an expression of the discomfort experienced during the manoeuvre, as in the case of functional eye blinking. The latter highlights the importance of adequate explanation and reassurance prior to performing a positional manoeuvre, particularly in individuals susceptible to anxiety. In all cases presented, the functional eye movements were largely asymptomatic and resolved either spontaneously (case 1), or with treatment of the underlying precipitant (cases 2, 4), without the need for specific interventions. Finally, a careful eye movement assessment in patients presenting with vertigo or imbalance necessitates a positional manoeuvre to correctly identify vestibular disorders, and associated functional signs, thus allowing appropriate treatment and avoiding unnecessary investigations.

## Electronic supplementary material

Below is the link to the electronic supplementary material.

Supplementary file1 Video 1 Videonystagmographic recording showing central positional downbeat nystagmus. At 00:24, the examiner verbally notes an apparent convergent effort, upon which convergence then begins (00:26), with associated pupillary constriction. (MP4 2775 kb)

Supplementary file2 Video 2 Following a head-hanging positional manoeuver, the patient develops a persistent eyelid blinking with eyeball elevation (Bell’s phenomenon) that is sustained for the duration of the manoeuvre, but not present during the remainder of the assessment or consultation. (MP4 2010 kb)

Supplementary file3 Video 3 Videonystagmographic recording showing bursts of rapid horizontal back-to-back oscillations associated with an excess of eyelid blinking. (MP4 7989 kb)

Supplementary file4 Video 4 Uniocular videonystagmographic recording showing torsional clockwise nystagmus (right posterior canal BPPV) followed by a rapid burst of horizontal eye oscillations without a slow phase eye movement (data not shown). (MP4 3864 kb)
